# School of Thrombectomy—A 3-Step Approach to Perform Acute Stroke Treatment with Simulator Training and Virtual Supervision by Remote Streaming Support (RESS)

**DOI:** 10.1007/s00062-022-01242-2

**Published:** 2022-12-15

**Authors:** Daniel Paech, Nils Lehnen, Asadeh Lakghomi, Arndt Schievelkamp, Christian Gronemann, Felix J. Bode, Alexander Radbruch, Franziska Dorn

**Affiliations:** 1 Clinic for Neuroradiology, University Hospital Bonn, Venusberg-Campus 1, 53127 Bonn, Germany; 2 Clinic for Neurology, University Hospital Bonn, Venusberg-Campus 1, 53127 Bonn, Germany

**Keywords:** Medical education, Telemedicine, Vascular intervention, Revascularization, Thrombectomy

## Abstract

As the number of neurointerventional procedures continues to increase, so does the need for well-trained neurointerventionalists. The purpose of this work was to establish and assess a systematic 3‑step approach to perform acute stroke treatment including simulator training and virtual supervision by remote streaming support (RESS). Five trainees (four men, one women) who have completed the 3‑step approach have answered an 11-item questionnaire (5-point Likert scale) in order to evaluate training step 1 (simulator). Furthermore, all trainees and one supervisor (female) answered a standardized questionnaire following the initial 15 consecutive thrombectomies for each trainee, corresponding to a total of 75 thrombectomies. The simulator training yielded learning benefits and confidence gain to perform MT on patients. The RESS approach facilitated the translation during the first independently performed thrombectomies on patients. In summary, the presented 3‑step approach increases the level of safety, as reported by the trainees and supervisor in this study and may enable an accelerated training of neurointerventionalists.

## Introduction

As the number of neurointerventional procedures performed continues to increase, so does the need for well-trained neurointerventionalists. Training to learn diagnostic examinations and thrombectomy procedures is a very time-consuming process faced with limited resources. Simulators have been used for years to help trainee interventionalists get started with neurointerventional procedures—without potentially harming the patient and without any unnecessary radiation exposure [1–5]. These approaches are followed by a series of supervised procedures, where the supervisor stands with the trainee at the angio table and can manually intervene at any time. Subsequently, the supervisor usually follows the procedures in the control room on the monitor and can give advice on it and, if necessary, enter the angio room and intervene in cases of emergency or severe technical difficulties.

Recently, smart angiography suites (SAS) have been introduced that feature bidirectional internet communication and collaboration for teleproctoring via audio and video technology [6–9]. These techniques facilitate direct support of (neuro) interventionalists that can be used in both elective and emergency settings [9, 10].

In order to increase the quality and make the duration of the training process as short and effective as possible, we have implemented a systematic curriculum including simulator training and virtual supervision by remote streaming support (RESS) at our institution. The curriculum comprises a 3-step systematic approach: during the initial phase, the trainee completes virtual thrombectomy (VT) training at the simulator. This step is followed by a series of onsite supervised interventions. Before performing neurointerventional procedures independently on their own responsibility, trainees complete the final phase where the supervisor is no longer physically present in the angio suite, but follows the entire procedure via a steerable camera system and audio communication in the SAS.

In this paper we aimed to evaluate this training concept by having five trainees and one supervisor independently answered questions based on a standardized questionnaire immediately after the interventions. The questionnaire assessed the feeling of safety during the individual steps of the intervention, the desire for active support during the procedure, and any procedural change based on the communication between supervisor and trainee. Finally, the VT approach (step 1 of our concept), was evaluated in terms of effectiveness and safety by the trainees after they have completed the curriculum.

## Methods

This study has been approved by the local ethics commitee and was carried out between June 2021 and September 2022. All data presented in this manuscript have been acquired in the course of quality assurance/quality improvement (QA/QI) measures at the Clinic for Neuroradiology, University Hospital Bonn, Germany.

### School of Thrombectomy: 3-Step Curriculum

**Fig. 1:**
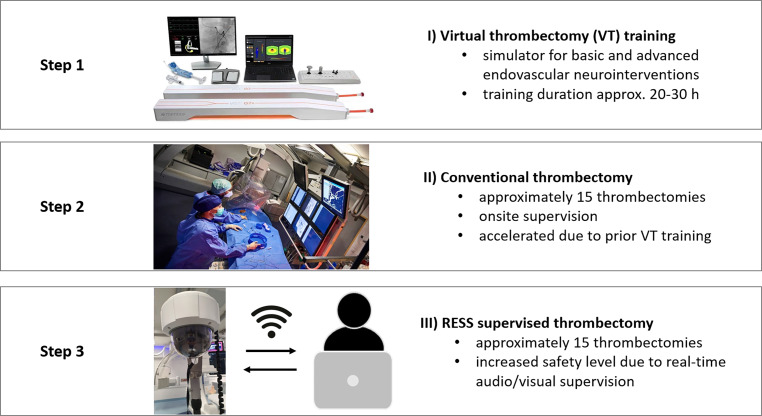
School of thrombectomy—a 3-step approach to perform acute stroke treatment. During *step* *1*, trainees perform virtual thrombectomies (*VT*) on an institution-owned simulator (Mentice Inc., IL, USA). *Step* *2* comprises a series of approximately 15 supervised neurointerventional procedures, where the supervisor stands at the angio table together with the trainee and can manually intervene at any time. Finally, the trainee completes approximately 15 mechanical thrombectomies with RESS (*step 3*). The RESS system provided by Tegus (Tegus Medical, Hamburg, Germany) is a low-latency, high-resolution solution for streaming live events both visually and auditorily

In June 2021, the 3‑step training curriculum for neurointerventionalists was established at our institution (Fig. 1). During step 1, trainees perform virtual thrombectomies (VT) on an institution-owned simulator (VIST G7, Mentice Inc., Gothenburg, Sweden). The training sessions at the simulator are initially supervised by an experienced neurointerventionalist. The total contact time during step 1 covers at least 25 h. Prior to and in parallel, all trainees were required to have performed 50 diagnostic angiographies independently and assisted in at least 20 thrombectomy procedures. Step 2 comprises a series of approximately 15 supervised thrombectomies, where the supervisor stands at the angio table together with the trainee and can manually intervene at any time. Finally, the trainee completes approximately 15 mechanical thrombectomies with RESS. The RESS system provided by Tegus (Tegus Medical, Hamburg, Germany) is a low-latency, high-resolution solution for streaming live events both visually and auditorily. The system includes a 360° rotatable, 90° tiltable, high-definition network camera (1920 × 1080 pixel resolution, 30 fps, 10 × optical zoom, 60 × digital zoom) placed on a transportable pentapod. The viewing angle, zoom and exposure functions can be remotely controlled by the supervisor. The treating interventionalist and the supervisor can communicate via a hands-free headset. Access to the platform requires a user account and an invitation to participate in the session, and the electronic protected health information (ePHI) is secured by encryption [6, 9].

### Evaluation of VT Training—11-Item Questionnaire

Five trainees (four men, one women) who have completed the 3‑step approach have answered an 11-item questionnaire in order to evaluate training step 1. Each question had to be answered on a Likert-scale with five items: 1 = full disagreement, 2 = partial disagreement, 3 = neutral/indifference, 4 = partial agreement, 5 = full agreement. The questionnaire had a special focus on the usability, the haptic feedback and authenticity, and the retrospectively evaluated learning benefit to perform mechanical thrombectomy (MT) in patients. The answers of all participants were visualized as scatter plots using MATLAB R2020a (The Mathworks, Natick, MA, USA). Furthermore, the median and mean ± standard deviation (std) values over all responses were calculated for each question.

### Evaluation of Thrombectomy Supervision by Remote Streaming Support (RESS)

Five trainees (all Clinic for Neuroradiology, University Hospital Bonn, Germany), who have completed the 3‑step approach answered a 21-item questionnaire following the first 15 consecutive MT performed with RESS support adding up to a total of 75 thrombectomies over all study participants. One proctor (F.D.) answered a similar 18-item questionnaire from the proctors’ perspective over 30 consecutive MT interventions with RESS. The questionnaires included questions that had to be answered on a 5-point Likert scale as well as yes-no questions. The questionnaires had a special focus on the personal feeling of safety during the different phases of the MT, confidence gain provided by the approach, as well as changes/adaptions in the process due to RESS feedback. The mean values ± standard deviation over the assessed MTs with RESS were visualized as bar graphs for each study participant. Furthermore, the reported confidence gain and the overall benefit were analyzed over time for each trainee. The resulting data points were fitted with an exponential function using MATLAB R2020a (The Mathworks).

## Results

### Evaluation of VT Training—10-Item Questionnaire

**Fig. 2:**
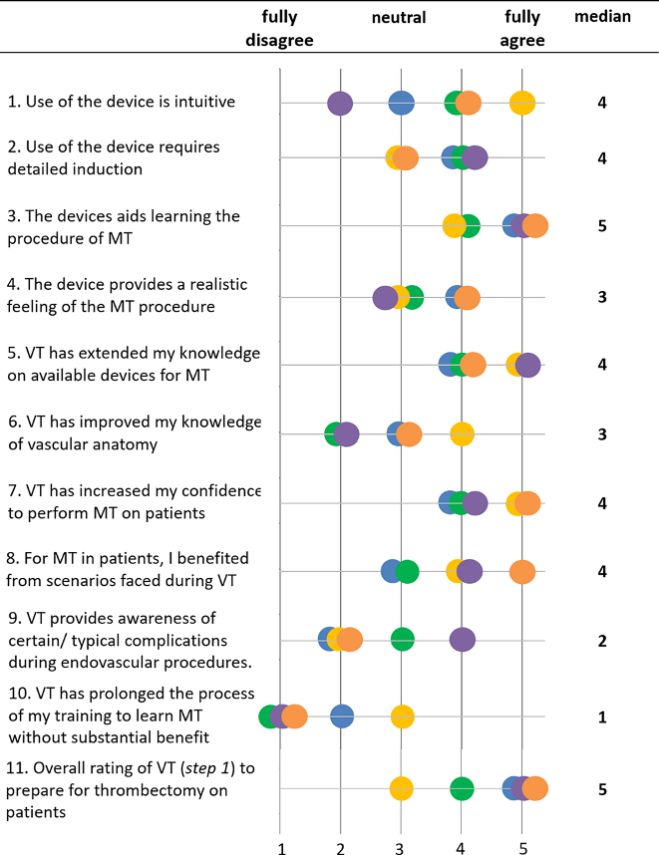
Evaluation of VT training—questionnaire results. Five trainees (each trainee *color coded*; four men, one women) who have completed the 3‑step approach have answered an 11-item questionnaire in order to evaluate training step 1. Each question had to be answered on a Likert-scale with five items: 1 = full disagreement, 2 = partial disagreement, 3 = neutral/indifference, 4 = partial agreement, 5 = full agreement. Median values overall are provided in the *right column* (*VT* virtual thrombectomy, *MT* mechanical thrombectomy)

The average duration of training hours during step 1 were 27.0 ± 2.45h over all trainees (range: minimum: 23 h, maximum 30 h). The results of the questionnaire for step 1 of the curriculum are provided in Fig. 2. There was a high overall agreement that the device aids learning MT (mean 4.60 ± 0.49, median 5), extends the knowledge on the available material repertoire for MT in patients (mean 4.40 ± 0.49, median 4), and that the technology is rather intuitive to use (mean 3.60 ± 1.0, median 4). Furthermore, the trainees reported an important confidence gain of simulated MT training as preparation for thrombectomies in real patients (mean 4.40 ± 0.49, median 4). Simulator training was retrospectively rated as moderately realistic (mean 3.40 ± 0.49, median 3). The trainees did not agree that VT training adequately prepared for possible complications and technical difficulties (mean 2.60 ± 0.8, median 2).

### Evaluation of Thrombectomy Supervision by Remote Streaming Support (RESS)

The trainees answered questionnaires for their first 15 consecutive MTs with RESS proctoring adding up to a total of 75 thrombectomies over all study participants. Extracranial stenting was performed in 8/75 interventions; intracranial stenting was performed in 2/75 cases.

**Fig. 3:**
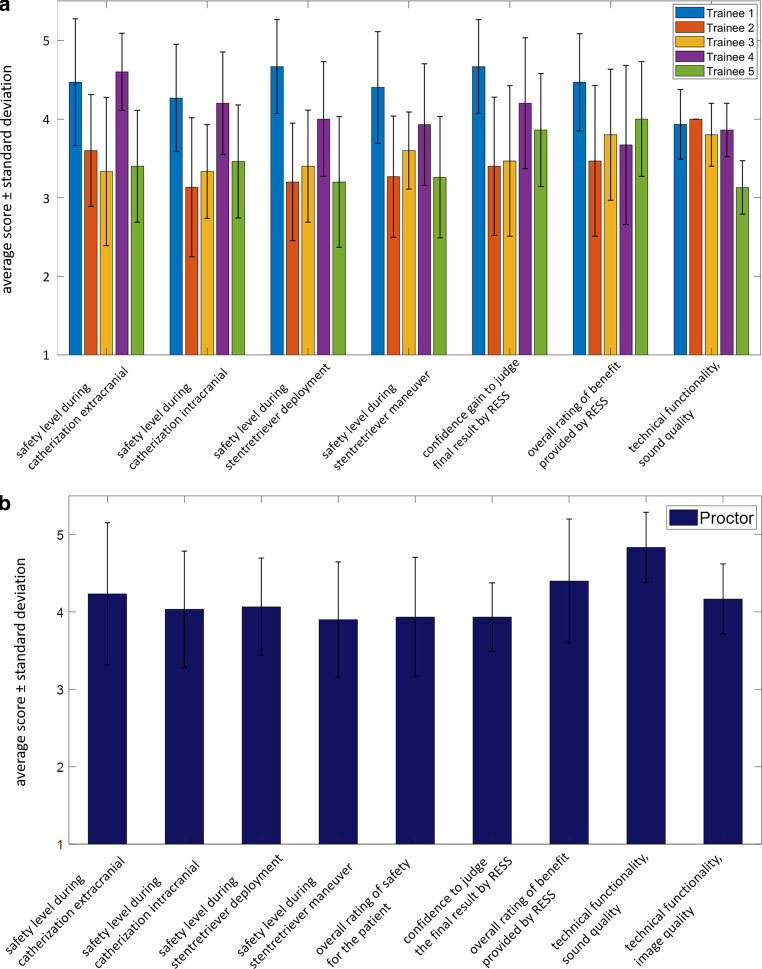
Evaluation of thrombectomy supervision by remote streaming support (*RESS*). Five trainees, who have completed the 3‑step approach answered a questionnaire following their first 15 consecutive MT performed with RESS (**a**). One proctor answered a similar questionnaire from the proctors’ perspective over 30 consecutive MT interventions (**b**). The questionnaires included 7 (**a**) and 9 (**b**) questions that had to be answered on a 5-point Likert scale. The evaluation specifically focused on the personal feeling of safety during the different phases of the MT, confidence gain provided by the approach, as well as changes/adaptions in the process due to RESS feedback. The mean values ± standard deviation are displayed as bar graphs for each study participant

Overall, a high level of safety was reported by the trainees during the first 15 independently performed thrombectomies during all steps (Fig. 3): extracranial catheterization (4.46 ± 0.81, 3.60 ± 0.71, 3.33 ± 0.94, 4.6 ± 0.49, 3.4 ± 0.71), intracranial catheterization (4.23 ± 0.68, 3.13 ± 0.88, 3.33 ± 0.60, 4.20 ± 0.65, 3.47 ± 0.72), and stent retriever deployment (4.67 ± 0.59, 3.20 ± 0.75, 3.40 ± 0.71, 4.00 ± 0.73, 3.20 ± 0.83), as well as retrieval maneuver (4.40 ± 0.71, 3.27 ± 0.77, 3.60 ± 0.49, 3.93 ± 0.77, 3.27 ± 0.77).

In particular, a benefit of audio-video supervision was seen during intracranial catheterization and the retrieval maneuver itself. Also, in the cases where extracranial stent PTA (8/75) and intracranial stent PTA (2/75) was necessary, the subjective level of safety was relatively high (extracranial PTA: 3.75 ± 0.43, 3.5 ± 0.50, 3.50 ± 0.50, two trainees: n/a, and intracranial stent PTA: 3.0 ± 0.00, 3.0 ± 0.00, three trainees: n/a). In total, trainees would have gladly accepted active manual assistance from the supervisor in nine situations: four times during extracranial catheterization, twice during intracranial catheterization, twice during the retrieval maneuver, and in one case during intracranial stent angioplasty.

**Fig. 4:**
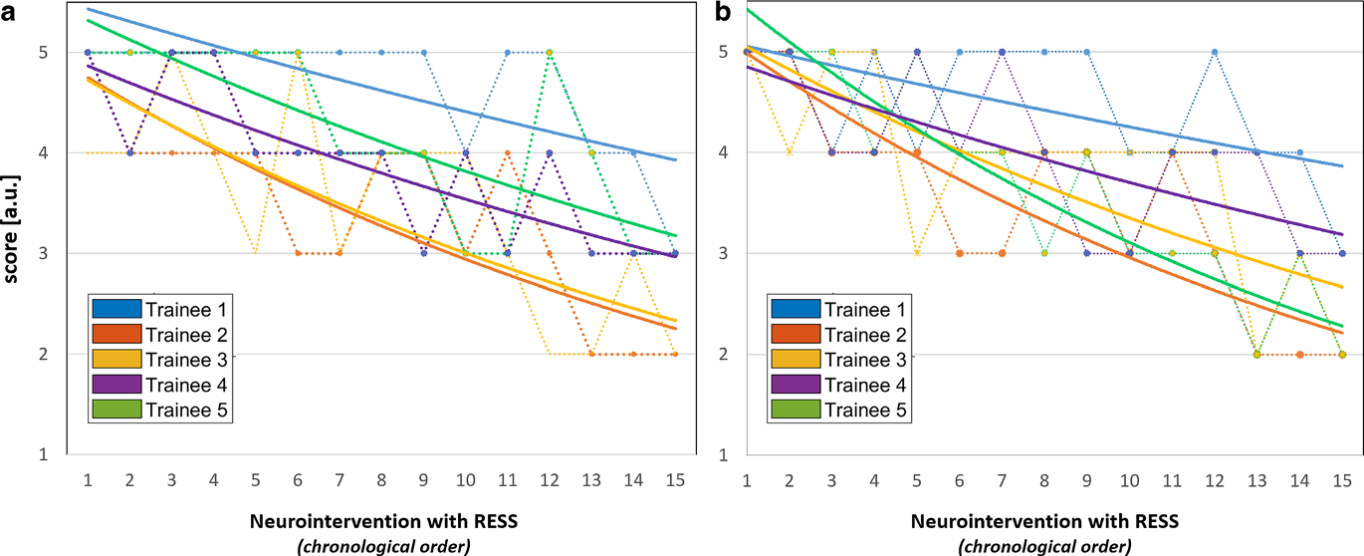
Confidence gain (**a**) and the overall benefit (**b**) provided by the RESS approach (step 3) over time. Data for the five enrolled trainees shown (*color coded*). The resulting data points were fitted with an exponential function (*dashed line*: raw data, *bold line*: exponential fit). A clear decay of benefit both in confidence gain and overall benefit was observed over time for all trainees. This trend indicates a continuously increasing degree of autonomy of all trainees

The supervising neurointerventionalist reported an overall high sense of safety with the desire to intervene in six situations: twice during extracranial catheterization, twice during intracranial catheterization, once during the retrieval maneuver, and one time during intracranial stenting. The trainees indicated that they actively changed their approach based on the advice of the supervisor in a total of 14 situations: 6 times during the extracranial, 4 times during the intracranial catheterization, and 2 times during the maneuver and in the 1 case where the decision was made for intracranial stent angioplasty. Fig. 4 visualizes the reported overall benefit of the RESS approach and confidence over time for each trainee. A clear decay of benefit was observed over time for all trainees indicating a continuously increasing degree of autonomy of the trainees.

## Discussion

In this work, we report a systematic 3‑step approach for accelerated and supervised thrombectomy training. The approach has been fully integrated into our neurointerventional training program. Enrolled trainees report learning benefits and confidence gain to perform MT on patients from simulator training. The RESS approach facilitates the translation during the first independently performed thrombectomies.

Since the first pivotal randomized trials were published in 2014 and integrated in guidelines, the number of thrombectomies performed has increased dramatically and so did the need for well-trained interventionists who can provide treatment 24/7 [11–14]. At the same time, the consequences of an inadequate training can be dramatic for patients: intracranial complications such as vascular injury, dissection, or thrombus occlusion are often fatal and require an immediate response from the treating physician. On the other hand, the level of technical challenge varies from patient to patient and depends on factors such as the individual vascular situation, localization and etiology of the vessel occlusion, as well as the potential need for stent angioplasty. Therefore, even experienced interventionalists are often confronted with new situations and the growing experience helps to react adequately here.

Simulation devices can prepare physicians in training to some degree for these challenges. Thus, a high training effect through simulation training could be demonstrated, especially for neurointerventional novices [1–4, 15]. These results are in line with our findings: all of our trainees rated the learning and practice effect of the initial simulator training at the end of the curriculum as very valuable. Simulation training has also been described to be one important factor in preventing technical and cognitive errors that may result in complications during neuroendovascular procedures [5, 16]. In this context, our study participants rather disagreed that the simulator approach provides awareness of typical complications during endovascular procedures. The overall benefit of simulation training to prepare for real thrombectomies was reported as very high by our trainees. All trainees stated that their extracranial and intracranial catheterization skills had improved because of the simulator training and that the simulator training had increased their confidence to perform thrombectomies by themselves.

A particular challenge during the process of becoming a neurointerventionalist is the transition from supervised thrombectomy (where the fact that an experienced colleague is present and can intervene at any time gives a feeling of safety), to performing the intervention independently and on one’s own responsibility. The latter often takes place outside normal working hours, which causes a certain inhibition to call in a more experienced colleague. Telemedical live support can help to simplify this step and increase the level of safety of the treating physician by incorporating audio-video technology and internet connectivity into the angio suite. The benefit of live telemedicine support has been described in other medical fields such as endoscopic or surgical procedures [17–20]. Overall, the trainees in our study agreed that the virtual presence of the supervisor and the possibility of unmediated communication had significantly improved their personal sense of security. All trainees indicated that they gained confidence from the virtual presence of the supervisor. While the trainees reported a very high benefit during the initial thrombectomies, it decreased over time for the consecutively performed thrombectomies. This finding may indicate the increasing experience and confidence gain of the trainees resulting in more autonomous procedures.

The COVID-19 pandemic has rapidly transformed the healthcare system and virtual techniques are playing an increasingly important role in both education and clinical practice; during the times of limited travel, the first-time use of new devices was supported remotely for the first time by a more experienced proctor, either through RESS or through traditional Skype [7, 21]. Smart glasses is another interesting new development that can be included into neurointerventional training: they offer a realistic impression while watching interventional cases for trainees and have already been described as a helpful tool to allow trainees to observe neurointerventional procedures in other centers [8].

In a monocentric report in which one neurointerventional trainee performed 10 consecutive procedures under live supervision and 10 comparable procedures under remote supervision conducted by a commercially available streaming system, the authors found no difference in contrast use, fluoroscopy time, or technical success [22].

Neurovascular diseases as well as their treatment are highly variable between different patients and present with individual challenges; therefore, it is difficult to define criteria that make any objective assessment of telemedical supervision of neurointerventional procedures possible; however, our data strongly suggest that the benefit is subjectively high, especially when beginning to perform thrombectomies independently.

This study had some limitations. The number of participants enrolled in the 3‑step approach is relatively low; however, the current study cohort (5 trainees, 1 proctor, a total of 75 thrombectomies with RESS) represents the largest sample size investigating a novel, systematic use of simulator training and RESS-guided acute stroke treatment. All participants of this study indicated that they subjectively gained safety through this structured 3‑step training concept. Objective criteria to quantify the success of the concept are difficult to define; however, this study may motivate forthcoming investigations of training neurointerventionalists using simulator devices and audio-visual supervision by RESS.

## Conclusion

The inclusion of both a simulator-training as well as a remote audio-visual supervision resulted in an increased level of safety for neuroradiologists in training who perform thrombectomies independently for the first time. Through the systematic 3‑step training concept, the safety of performance can be gradually increased, which in turn potentially benefits patient safety and treatment effectiveness. The benefit can potentially be transferred to other neurointerventional procedures, such as aneurysm treatment or embolization.

## References

[1] Kreiser K, Gehling K, Zimmer C (2019). Simulation in Angiography—Experiences from 5 Years Teaching, Training, and Research. Rofo.

[2] Kreiser K, Ströber L, Gehling KG, Schneider F, Kohlbecher S, Schulz CM (2021). Simulation training in neuroangiography—validation and effectiveness. Clin Neuroradiol.

[3] Fargen KM, Siddiqui AH, Veznedaroglu E, Turner RD, Ringer AJ, Mocco J (2012). Simulator based angiography education in neurosurgery: results of a&nbsp;pilot educational program. J NeuroIntervent Surg.

[4] Davids J, Manivannan S, Darzi A, Giannarou S, Ashrafian H, Marcus HJ (2021). Simulation for skills training in neurosurgery: a&nbsp;systematic review, meta-analysis, and analysis of progressive scholarly acceptance. Neurosurg Rev.

[5] Liebig T, Holtmannspötter M, Crossley R, Lindkvist J, Henn P, Lönn L (2018). Metric-based virtual reality simulation: a&nbsp;paradigm shift in training for mechanical thrombectomy in acute stroke. Stroke.

[6] von Hessling A, Del Castillo RT, Roos JE, Karwacki GM (2022). Technical considerations and tips for using the Tegus remote proctoring system in elective and emergency cases and in webinars. J Neurointerv Surg.

[7] Ricci DR, Marotta TR, Riina HA, Wan M, De Vries J (2016). A training paradigm to enhance performance and safe use of an innovative neuroendovascular device. J Mark Access Health Policy.

[8] Martínez-Galdámez M, Fernández JG, Arteaga MS, Pérez-Sánchez L, Arenillas JF, Rodríguez-Arias C (2021). Smart glasses evaluation during the COVID-19 pandemic: First-use on Neurointerventional procedures. Clin Neurol Neurosurg.

[9] Bechstein M, Buhk J-H, Frölich AM, Broocks G, Hanning U, Erler M (2021). Training and supervision of thrombectomy by remote live streaming support (RESS). Clin Neuroradiol.

[10] Lim DZ, Mitreski G, Maingard J, Kutaiba N, Hosking N, Jhamb A, Ranatunga D, Kok HK, Chandra RV, Brooks M, Barras C, Asadi H (2022). The smart angiography suite. J Neurointerv Surg.

[11] Berkhemer OA, Fransen PS, Beumer D, Van Den Berg LA, Lingsma HF, Yoo AJ (2015). A randomized trial of intraarterial treatment for acute ischemic stroke. N Engl J Med.

[12] Goyal M, Menon BK, van Zwam WH, Dippel D, Mitchell P, Demchuk A, HERMES collaborators (2016). Endovascular thrombectomy after large-vessel ischaemic stroke: a&nbsp;meta-analysis of individual patient data from five randomised trials. Lancet.

[13] Smith EE, Saver JL, Cox M, Liang L, Matsouaka R, Xian Y (2017). Increase in endovascular therapy in get with the guidelines-stroke after the publication of pivotal trials. Circulation.

[14] Lee JV, Scott A, Osbun J, Zipfel G (2021). Impact of stroke call on career satisfaction and burnout for academic neurointerventionalists: a grounded theory model. World Neurosurg.

[15] Paech D, Giesel FL, Unterhinninghofen R, Schlemmer H-P, Kuner T, Doll S (2017). Cadaver-specific CT scans visualized at the dissection table combined with virtual dissection tables improve learning performance in general gross anatomy. Eur Radiol.

[16] Ospel JM, Kashani N, Mayank A, Cimflova P, Heran M, Pandey S (2020). Impact and prevention of errors in endovascular treatment of unruptured intracranial aneurysms. Interv Neuroradiol.

[17] Burgess CLP, Syms MJ, Holtel CMR, Birkmire-Peters DP, Johnson MRE, Ramsey MMJ (2002). Telemedicine: teleproctored endoscopic sinus surgery. Laryngoscope.

[18] Ereso AQ, Garcia P, Tseng E, Gauger G, Kim H, Dua MM (2010). Live transference of surgical subspecialty skills using telerobotic proctoring to remote general surgeons. J Am Coll Surg.

[19] Datta N, MacQueen IT, Schroeder AD, Wilson JJ, Espinoza JC, Wagner JP (2015). Wearable technology for global surgical teleproctoring. J Surg Educ.

[20] Suzuki M, Vyskocil E, Ogi K, Matoba K, Nakamaru Y, Homma A, Wormald PJ, Psaltis AJ (2021). Remote Training of Functional Endoscopic Sinus Surgery With Advanced Manufactured 3D Sinus Models and a Telemedicine System. Front Surg.

[21] Bechstein M, Elsheikh S, Wodarg F, Taschner CA, Hanning U, Buhk J-H (2020). Interhospital teleproctoring of endovascular intracranial aneurysm treatment using a&nbsp;dedicated live-streaming technology: first experiences during the COVID-19 pandemic. BMJ Case Rep.

[22] Hassan AE, Desai SK, Georgiadis AL, Tekle WG (2022). Augmented reality enhanced tele-proctoring platform to intraoperatively support a neuro-endovascular surgery fellow. Interv Neuroradiol.

